# Development and Validation of a Gene Mutation-Associated Nomogram for Hepatocellular Carcinoma Patients From Four Countries

**DOI:** 10.3389/fgene.2021.714639

**Published:** 2021-09-21

**Authors:** Tingping Huang, Tao Yan, Gonghai Chen, Chunqing Zhang

**Affiliations:** ^1^ Department of Gastroenterology, Shandong Provincial Hospital, Cheeloo College of Medicine, Shandong University, Jinan, China; ^2^ Department of Thoracic Surgery, Shandong Provincial Hospital, Cheeloo College of Medicine, Shandong University, Jinan, China

**Keywords:** TCGA, ICGC, hepatocellular carcinoma, nomogram, immunotherapy

## Abstract

**Background:** Genomic alteration is the basis of occurrence and development of carcinoma. Specific gene mutation may be associated with the prognosis of hepatocellular carcinoma (HCC) patients without distant or lymphatic metastases. Hence, we developed a nomogram based on prognostic gene mutations that could predict the overall survival of HCC patients at early stage and provide reference for immunotherapy.

**Methods:** HCC cohorts were obtained from The Cancer Genome Atlas (TCGA) and International Cancer Genome Consortium (ICGC) databases. The total patient was randomly assigned to training and validation sets. Univariate and multivariate cox analysis were used to select significant variables for construction of nomogram. The support vector machine (SVM) and principal component analysis (PCA) were used to assess the distinguished effect of significant genes. Besides, the nomogram model was evaluated by concordance index, time-dependent receiver operating characteristics (ROC) curve, calibration curve and decision curve analysis (DCA). Gene Set Enrichment Analysis (GSEA), CIBERSORT, Tumor Immune Dysfunction and Exclusion (TIDE) and Immunophenoscore (IPS) were utilized to explore the potential mechanism of immune-related process and immunotherapy.

**Results:** A total of 695 HCC patients were selected in the process including 495 training patients and 200 validation patients. Nomogram was constructed based on T stage, age, country, mutation status of DOCK2, EYS, MACF1 and TP53. The assessment showed the nomogram has good discrimination and high consistence between predicted and actual data. Furthermore, we found T cell exclusion was the potential mechanism of malignant progression in high-risk group. Meanwhile, low-risk group might be sensitive to immunotherapy and benefit from CTLA-4 blocker treatment.

**Conclusion:** Our research established a nomogram based on mutant genes and clinical parameters, and revealed the underlying association between these risk factors and immune-related process.

## Introduction

Liver cancer is one of the most common cancers in the world, according to recent global estimates, liver cancer ranks sixth in incidence rate and fourth in mortality (Bray et al., 2018). In particular, hepatocellular carcinoma (HCC) accounts for 75–85% of all cases of liver cancer (Bray et al., 2018). Liver cancer has a disproportionate impact on the global poor, and as economic gaps widen, the mortality rate of liver cancer in poor areas is predicted to rise by 40% (Siegel et al., 2019). Developed countries have begun to consider HCC a high priority public health concern, because its risk factors, such as alcohol consumption and obesity, are increasingly common as lifestyles change (Makarova-Rusher et al., 2016; Baecker et al., 2018). Therefore, it is an urgent need to improve tools for clinical diagnoses and assessments of prognoses early in the course of HCC. While these tools focus on biological factors, it is also important to consider socioeconomic and lifestyle influences when analyzing HCC patient prognoses (Tsai et al., 2018).

The progression of hepatic carcinogenesis involves a variety of factors, including environmental exposure, somatic mutations and transcriptional or epigenetic variations (Makarova-Rusher et al., 2016; Liu et al., 2018; Calderaro et al., 2019). Genetic mutations are of particular importance. There are approximately 30,000 genes in human cells, and these genes serve as the targets of the many genetic mutation events that occur over the course of a human life. Considering the potentially astronomical number of potential mutation events, screening for significant mutations has long been a hot research topic (Rao et al., 2017).

This research has led to the identification of some key mutations, which commonly act as therapeutic targets and frequently have particular significance to specific cancers. For example, gefitinib, erlotinib, and afatinib are potent targeted agents that are used for the treatment of advanced non-small cell lung cancers in which the gene coding for the epidermal growth factor receptor (EGFR) has been mutated (Mitsudomi et al., 2010; Wu et al., 2017). Similarly, trastuzumab and pertuzumab are targeted to breast cancers in which another growth factor receptor, HER2, is aberrantly overproduced (Swain et al., 2015; Swain et al., 2020). In addition to serving as therapeutic targets, some gene mutations are also effective prognostic indicators of patient outcomes. Examples in this regard include mutations in TP53, PTEN and RB1 in prostate cancer and mutations in TP53, PIK3CA, ERBB2 and KRAS in gastric adenocarcinoma (Kato et al., 2018; Hamid et al., 2019). However, prognostic tools for HCC patients based on specific gene mutations have not been well established.

When studying mutations in tumors, it is important to consider the tumor mutation burden (TMB), which is defined as the total number of mutations, including base substitutions, gene insertions and deletions, per tumor genomic region (Fancello et al., 2019). A higher TMB means that the cancerous cells generate more new antigens that will be easily recognized by immune cells (Steuer and Ramalingam, 2018). Patients with high TMB values have been found to be sensitive to treatment with immune checkpoint inhibitors (ICIs) in the context of lung cancer, melanoma and urothelial carcinoma (Chan et al., 2015; Powles et al., 2018; Reck et al., 2021). The advent of ICIs was a milestone event for treatment of advanced tumor and immunotherapy had no advantage over conventional therapy if excluding ICIs (Zhu et al., 2017; Petrelli et al., 2021a). Compared with chemotherapy, ICIs treatment was safer and induced less infection for patients with solid tumors (Petrelli et al., 2021b). In terms of applicable population, recent evidence indicated that patients aged more than 75 years still benefited from ICIs (Petrelli et al., 2021a). Although the ICIs has shown some benefit in clinical therapy, it is cautious that efficiency of each ICIs in different tumors was various. For example, non-small cell lung cancer patients possessed slightly better prognoses under anti-PD-1therapy than anti-PD-L1therapy (Tartarone et al., 2019). The correlation of TMB with ICIs treatment success indicates that it is feasible and meaningful to explore tumor mutations in order to guide clinical choices involving immunotherapy.

While TMB has shown predictive power in the treatment and prognosis of several cancer types, it has been insufficiently applied to HCC. It is critical, then, to develop a multi-dimensional model to identify patients at high risk in order to facilitate personalized medicine in HCC patients. In the present work, we analyzed representative mutated genes in HCC cases without distant or lymphatic metastases. Conventional risk assessment was based mainly on tumor, lymph node and metastasis (TNM) staging, which ignores the biological heterogeneity of the primary tumor (Kee et al., 2013; Buonaguro, 2020). We screened The Cancer Genome Atlas (TCGA) and International Cancer Genome Consortium (ICGC) databases to identify significant genes to construct a prognostic nomogram and to study the correlation between the derived risk score and tumor immunology, including immune cell infiltration propensity and predicted sensitivity to treatment with ICIs. Besides, the machine learning algorithms were used to evaluate the application of nomogram.

## Materials and Methods

### Data Collection

Transcriptome profile data, single nucleotide variation data and corresponding clinical data were downloaded from the TCGA data portal (https://cancergenome.nih.gov/) and ICGC data portal (https://icgc.org/). As TCGA and ICGC data are open to the public, approval from a local ethics committee is not necessary. Inclusion criteria included: 1) complete clinical information; 2) complete survival data; 3) complete gene mutation data; 4) a single primary tumor lesion; and 5) no distant or lymphatic metastases. In total, we identified five cohorts containing 695 patients, who came from four countries. Given that the incidence and mortality of HCC are influenced by lifestyle and socioeconomic factors (Makarova-Rusher et al., 2016; Baecker et al., 2018), we decided to include nationality as a prognostic factor in the predictive model. To achieve this vision and to ensure a balanced distribution of countries between training and validation sets, we randomly divided the patients in each cohort into two groups according to the ratio of 7:3. Then, we extracted 70% of patients of each cohort to form the training set, which contained a total of 495 patients, and 30% of patients to form the validation set, which contained 200 patients. The R code is provided in Supplementary Materials.

### Processing of Variables

Continuous variables were converted into categorical variables according to a linear assumption. Age was divided based on the optimal cut-off value generated by X-tile software version 3.6.1 (Yale University School of Medicine, United States). Overall survival (OS) was the primary endpoint in this study.

### Establishment and Validation of the Nomogram

A univariate Cox analysis was used to identify significant variables, as defined by a *p* value less than 0.05, from the clinical information and gene mutation data. A multivariate Cox analysis was then utilized to further identify significant variables to construct the nomogram. We classified the patients into a high-risk group and a low-risk group, with the cutoff value defined as the median of the risk score (0.9180). The related R code is provided in Supplementary Materials. A Kaplan-Meier curve analysis was applied to calculate patient OS.

Validation was performed using concordance index (C-index), time-dependent receiver operating characteristics (ROC) curve, calibration curve and decision curve (DCA) analyses. A C-index was used to assess the discrimination of the nomogram: the higher the C–index, the more accurate the survival prediction. Calibration plots were utilized to compare predictions based on the nomogram with actual outcomes. ROC curve analysis was applied to determine the sensitivity and specificity of the nomogram, and DCA was used to measure the efficiency of the nomogram. R software version 3.6.3, with packages limma, survival, survminer, rms, foreign and survivalROC, was used for all analysis. Differences were considered statistically significant with *p* < 0.05.

### Evaluation via Machine Learning

The SVM, one of the supervised learning models, was performed via “Skelearn” under python 3.9.5 environment. We chose the “rbf” as kernel for SVM and adjusted the parameters, such as gamma and class weight, to make sure SVM performs optimally. The transcriptome profile data of TCGA was divided randomly into training and validation group. Based on the selected genes, SVM was used to distinguish the normal and carcinoma tissue. The relative code was provided in the Supplementary Materials. Meanwhile, we used an unsupervised learning algorithm called principal component analysis (PCA) to reduce the dimensionality of the nomogram so that the spatial distribution of the sample could be visualized.

### Functional Enrichment Analysis

Data from total of 219 HCC patients from the TCGA cohort with complete RNA-seq data were used for functional prediction. Gene set enrichment analysis (GSEA) was employed as a computational method that explores whether a defined set of genes shows statistically significant differences between two biological states (Subramanian et al., 2005). To investigate the main biological functions and signaling pathways of the risk score group, gene ontology (GO) and Kyoto Encyclopedia of Genes and Genomes (KEGG) analyses were performed using GSEA v4.1.0 software. After performing 1,000 permutations, gene sets with a nominal *p* value < 0.05 were considered statistically significant.

### Evaluation of Immune Cell Status

In total, 219 HCC patients with complete RNA-seq data from the TCGA cohort were used for CIBERSORT analysis, which is an approach to characterizing the composition of 22 different tumor-infiltrating lymphocyte subsets within specific tissues based on their gene expression profiles (Newman et al., 2015). To uncover the underlying mechanisms relating gene mutations to immune cell status, we estimated the abundance of immune cell infiltration in HCC patients without distant and lymphatic metastases on the basis of the CIBERSORT algorithm.

### Prediction of Response to ICIs Treatment

There are 12 published clinical studies of ICIs and eight published CRISPR screens on the Tumor Immune Dysfunction and Exclusion (TIDE) website developed by Harvard University. This website explores two primary mechanisms of tumor immune evasion, T cell dysfunction and T cell exclusion, to evaluate the tumor microenvironment and to predict responses to treatment with ICIs (Fu et al., 2020). The TIDE score, T cell dysfunction score, and T cell exclusion score of HCC patients from the TCGA dataset were retrieved from the TIDE website (http://tide.dfci.harvard.edu/) after uploading the transcriptome profiles of 219 HCC patients from TCGA.

### Assessment of Choice of Specific ICIs Treatment

An immunophenoscore (IPS) was used to represent tumor immunogenicity on a scale from 0 to 10. It has been confirmed that an IPS value is positively associated with tumor immunogenicity and predicts a patient’s response to ICIs treatment (Charoentong et al., 2017). Therefore, we obtained the IPS score of 219 HCC patients from The Cancer Immunome Atlas (https://tcia.at/) to compare the potential use of immunotherapy in high- and low-risk score groups.

## Results

### Baseline Information of HCC Patients

**FIGURE 1:**
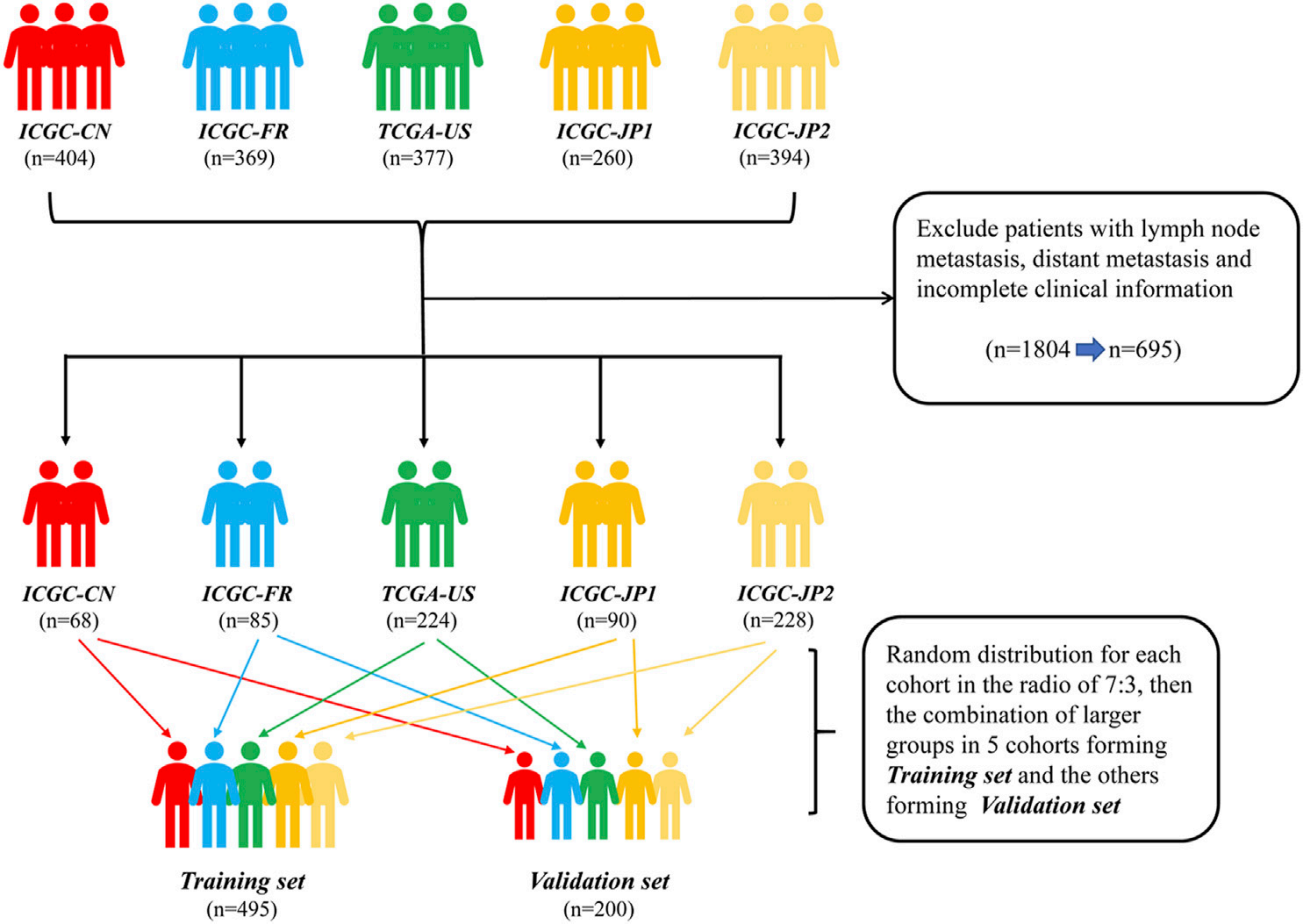
Flow diagram of the HCC patients without distant and lymphatic metastasis in training and validation sets. ICGC-CN: LICA-CN, liver cancer-China; ICGC-FR: LICA-FR, liver cancer-France; TCGA-US: TCGA-LIHC, The Cancer Genome Atlas-liver hepatocellular carcinoma; ICGC-JP1: LIRI-JP, liver cancer RIKEN-Japan; ICGC-JP2: LINC-JP, liver cancer NCC-Japan., hepatocellular carcinoma.

A total of 695 patients were selected from five HCC data sets: Liver Cancer-China (LICA-CN), Liver Cancer-France (LICA-FR), Liver Cancer NCC-Japan (LINC-JP), Liver Cancer RIKEN-Japan (LIRI-JP), and TCGA-Liver Hepatocellular Carcinoma (TCGA-LIHC) (Figure 1). Among them, 495 patients were utilized as a training set to establish the predictive nomogram. The remaining 200 patients were used to validate the nomogram. The baseline characteristics of these 695 HCC patients, who did not have distant or lymphatic metastases, are shown in Table 1. Overall, 512 (73.7%) patients were male. Together, Japanese and American patients (*n* = 542; 78.0%) accounted for the majority of the subjects. Patients were distributed into groups based on age (less than 47, between 47 and 72, and greater than 72 years); 471 (67.8%) of the patients were between 47 and 72 years old. The distribution of variables between the training set and validation set was well balanced, with all *p* values greater than 0.05.

**TABLE 1:** Clinical characteristics of all 695 hepatocellular carcinoma patients.

Variables	All patients,n (%)	Training set,n (%)	Validation set,n (%)	*p* value
Total	695 (100.0)	495 (71.2)	200 (28.8)	
Sex				0.163
Female	183 (26.3)	123 (24.8)	60 (30.0)	
Male	512 (73.7)	372 (75.2)	140 (70.0)	
Age				0.601
<47	73 (10.5)	53 (10.7)	20 (10.0)	
47–72	471 (67.8)	330 (66.7)	141 (70.5)	
>72	151 (21.7)	112 (22.6)	39 (19.5)	
T stage				0.392
I	201 (28.9)	151 (30.5)	50 (25.0)	
II	301 (43.3)	214 (43.2)	87 (43.5)	
III	185 (26.6)	125 (25.3)	60 (30.0)	
IV	8 (1.2)	5 (1.0)	3 (1.5)	
Country				0.998
China	68 (9.8)	48 (9.7)	20 (10.0)	
France	85 (12.2)	61 (12.3)	24 (12.0)	
Japan	318 (45.8)	226 (45.7)	92 (46.0)	
American	224 (32.2)	160 (32.3)	64 (32.0)	

### Identification of Prognostic Variables Based on Cox Regression Analysis

Four clinical variables and 116 commonly mutated genes (Supplementary Table S1) were selected to conduct a univariate Cox analysis (Table 2). The selected mutated genes represented a combination of the 50 most frequently mutated genes from the five study cohorts. We found that age, T stage, country and mutation status of five genes, TP53, MACF1, EYS, DOCK2 and FREM2, were significantly associated with OS in HCC patients without distant or lymphatic metastasis. Next, a multivariate Cox regression analysis illustrated that age, T stage, country and mutation status of four of the genes, TP53, MACF1, EYS and DOCK2, were independent from other factors and could be used as independent prognostic factors to establish a nomogram (Table 2).

**TABLE 2:** Univariate and multivariate analysis of overall survival for patients in the training set (495).

Variables	Total (%)	Univariate analysis		Multivariate analysis	
		HR (95%CI)	*p* value	HR (95%CI)	*p* value
Sex					
Female	123 (24.8)	reference	0.602		
Male	372 (75.2)	0.900 (0.604–1.340)			
Age					
<47	53 (10.7)	reference	0.043	reference	0.020
47–72	330 (66.7)	1.569 (0.811–3.033)	0.181	1.665 (0.849–3.266)	0.138
>72	112 (22.6)	2.279 (1.125–4.617)	0.022	2.168 (1.252–5.475)	0.011
T stage					
I	151 (30.5)	reference	<0.001	reference	<0.001
II	214 (43.2)	1.172 (0.740–1.857)	0.499	1.512 (0.910–2.512)	0.111
III	125 (25.3)	2.197 (1.374–3.513)	0.001	2.628 (1.594–4.333)	<0.001
IV	5 (1.0)	8.696 (3.027–24.976)	<0.001	5.637 (1.870–16.994)	0.002
Country					
China	48 (9.7)	reference	0.003	reference	0.001
France	61 (12.3)	1.643 (0.842–3.204)	0.145	1.666 (0.821–3.380)	0.157
Japan	226 (45.7)	0.702 (0.374–1.319)	0.272	0.628 (0.324–1.215)	0.167
American	160 (32.3)	1.379 (0.757–2.512)	0.293	1.442 (0.750–2.770)	0.272
TP53					
Wild	370 (74.7)	reference	0.038	reference	0.028
Mutation	125 (25.3)	1.497 (1.022–2.193)		1.565 (1.051–2.331)	
MACF1					
Wild	472 (95.4)	reference	0.040	reference	0.010
Mutation	23 (4.6)	2.038 (1.033–4.023)		2.536 (1.247–5.158)	
EYS					
Wild	466 (94.1)	reference	0.030	reference	0.019
Mutation	29 (5.9)	0.212 (0.052–0.857)		0.185 (0.045–0.756)	
DOCK2					
Wild	475 (96.0)	reference	0.001	reference	0.001
Mutation	20 (4.0)	3.312 (1.861–5.896)		2.797 (1.497–5.225)	
FREM2					
Wild	475 (96.0)	reference	0.022	reference	0.493
Mutation	20 (4.0)	2.217 (1.124–4.374)		1.298 (0.615–2.739)	

HR, hazard ratio; CI, confidence interval.

### The Construction and Validation of the Nomogram

**FIGURE 2:**
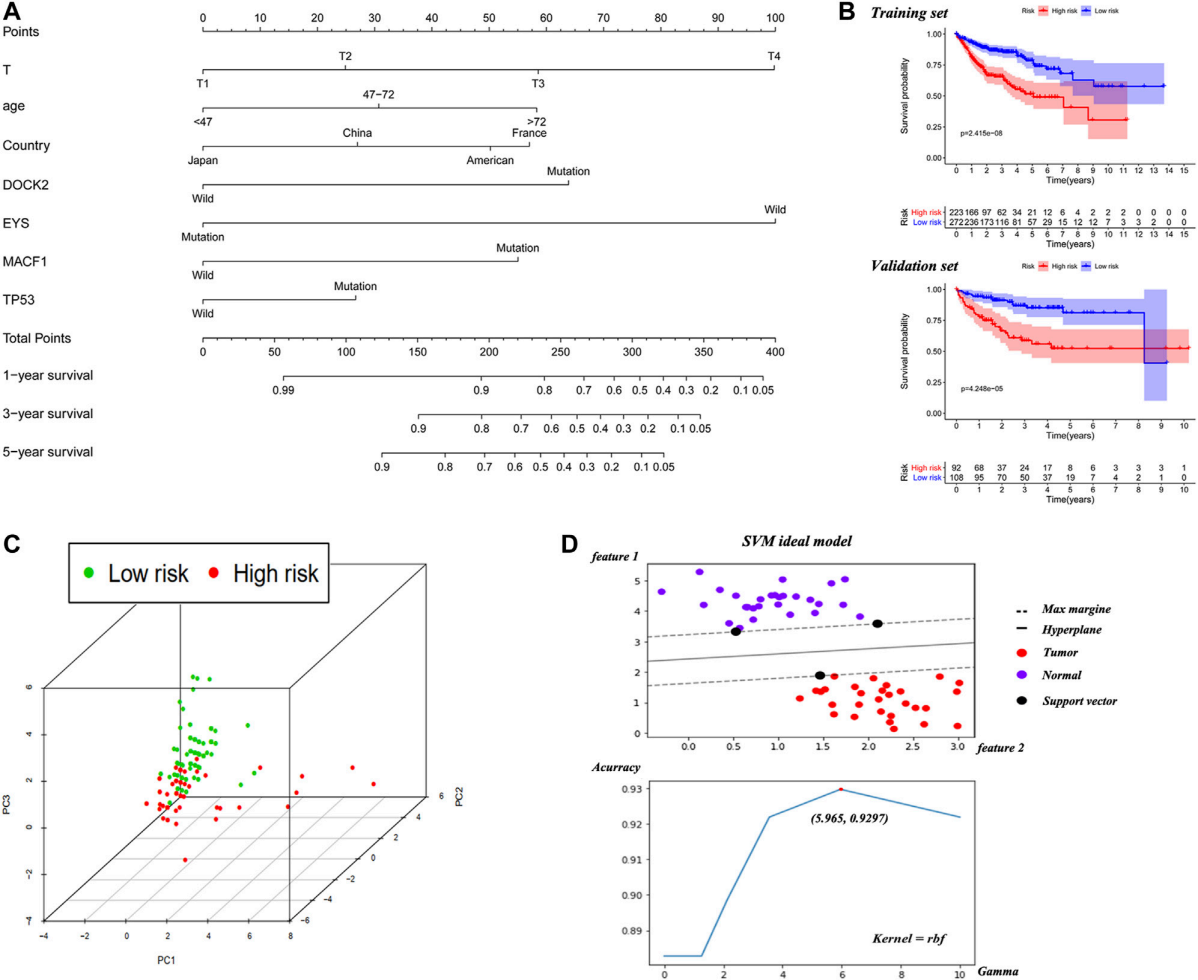
The nomogram based on training cohort and the efficient evaluation of training and validation cohort. **(A)** Nomogram predicting 1-year, 3-years and 5-years OS for HCC patients without distant and lymphatic metastasis. Each variable has a corresponding score on the point scale. Sum the score and locate it on the total point axis. Then, draw a vertical line down to get the nomogram-predicted probability at each time point. **(B)** Kaplan-Meier curve of high-risk and low-risk HCC patients based on the median of nomogram risk score in the training and validation cohort. **(C)** The visualization of high- and low-risk patients’ distribution via PCA algorithm. **(D)** The schematic diagram of SVM and the adjusting progression to solve maximum accuracy of the model.

A nomogram was established based on the noted parameters, including age, T stage, country and mutation status of TP53, MACF1, EYS and DOCK2 (Figure 2A). As shown in the nomogram, each risk factor is associated with a specific score. The scores for these risk factors are summed to produce a total score, which can be compared to a corresponding OS rate. Scores calculated in this way are predictive of 1-year, 3-years and 5-years survival rates. The Kaplan-Meier curve showed that the low-risk group is associated with a better OS than the high-risk group in both training and validation sets (Figure 2B).

To make the samples distribution visible, PCA algorithm was used to reduce the dimensionality of the nomogram and the 3D scatter diagram showed that the patients in high risk and low risk group from validation cohort were divided into two well-defined clusters (Figure 2C), which identified the broad applicability of this prediction model. Based on TP53, MACF1, EYS and DOCK2, the SVM constructed the hyperplane to distinguish the normal and cancer tissue and the highest accuracy was 92.97% when gamma was 5.965 (Figure 2D). During the progression of adjusting parameter, the accuracy, recall ratio and Area Under Curve (AUC) were throughout more than 0.8 (Supplementary Table S2). The precise diagnostic capability of SVM indicated those four genes might play important roles during the occurrence of liver cancer.

**FIGURE 3:**
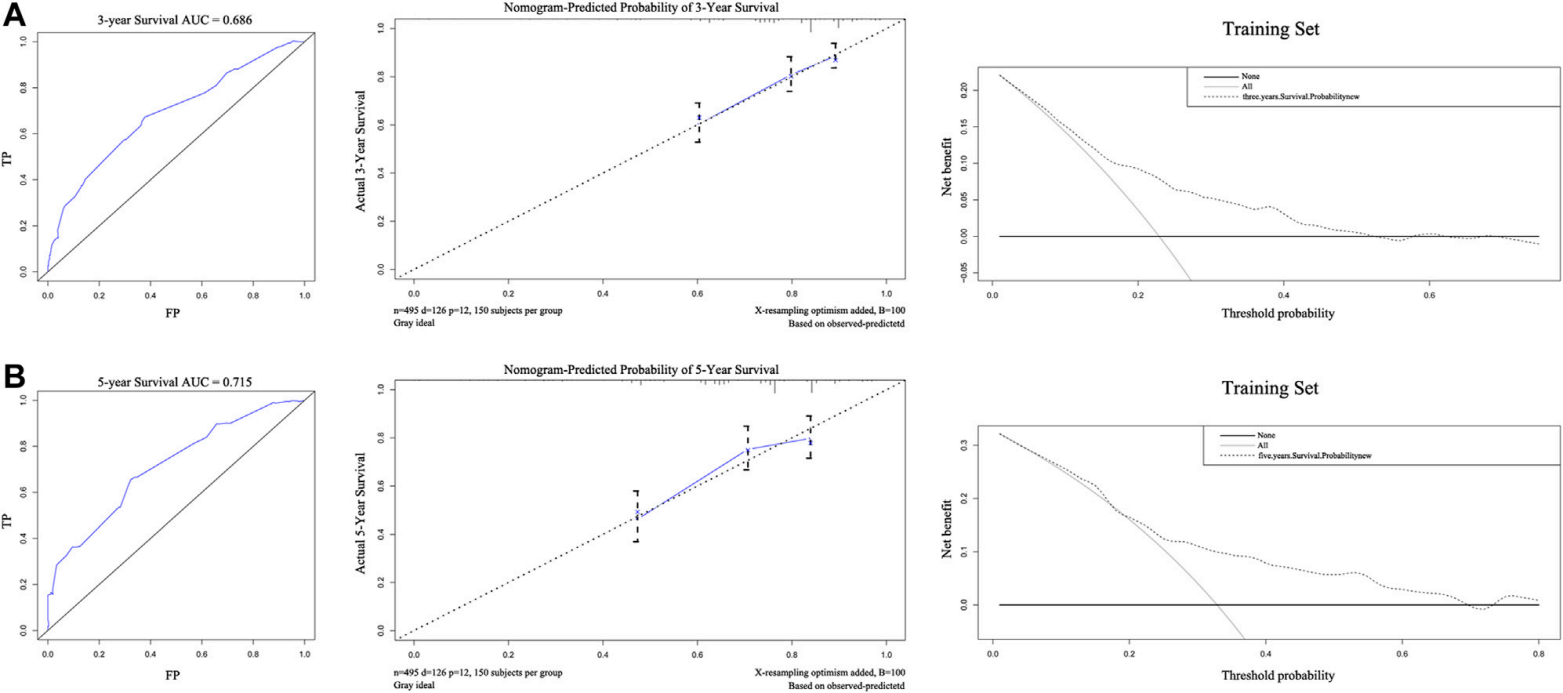
The areas under the receiver operating curves, Calibration plots, and decision curve analysis of OS associated nomograms in training set. The training set of 3-years **(A)** and 5-years **(B)**, respectively.

The C-index of the nomogram was 0.710, indicating strong predictive power. As shown in Figures 3A,B, the ROC curves of 3-years and 5-years survival rates were 0.686 and 0.715, respectively. Further supporting the quality of the nomogram, it was concluded that the actual and predicted survival are in good agreement according to the 3-years and 5-years calibration curves. Importantly, DCA of 3-years and 5-years survival rates demonstrated that within threshold probability ranges of approximately 0.1–0.6 and 0.2–0.8, respectively, nomogram-mediated intervention provides more net benefit to patients than is received in treat-all or treat-none patient schemes.

**FIGURE 4:**
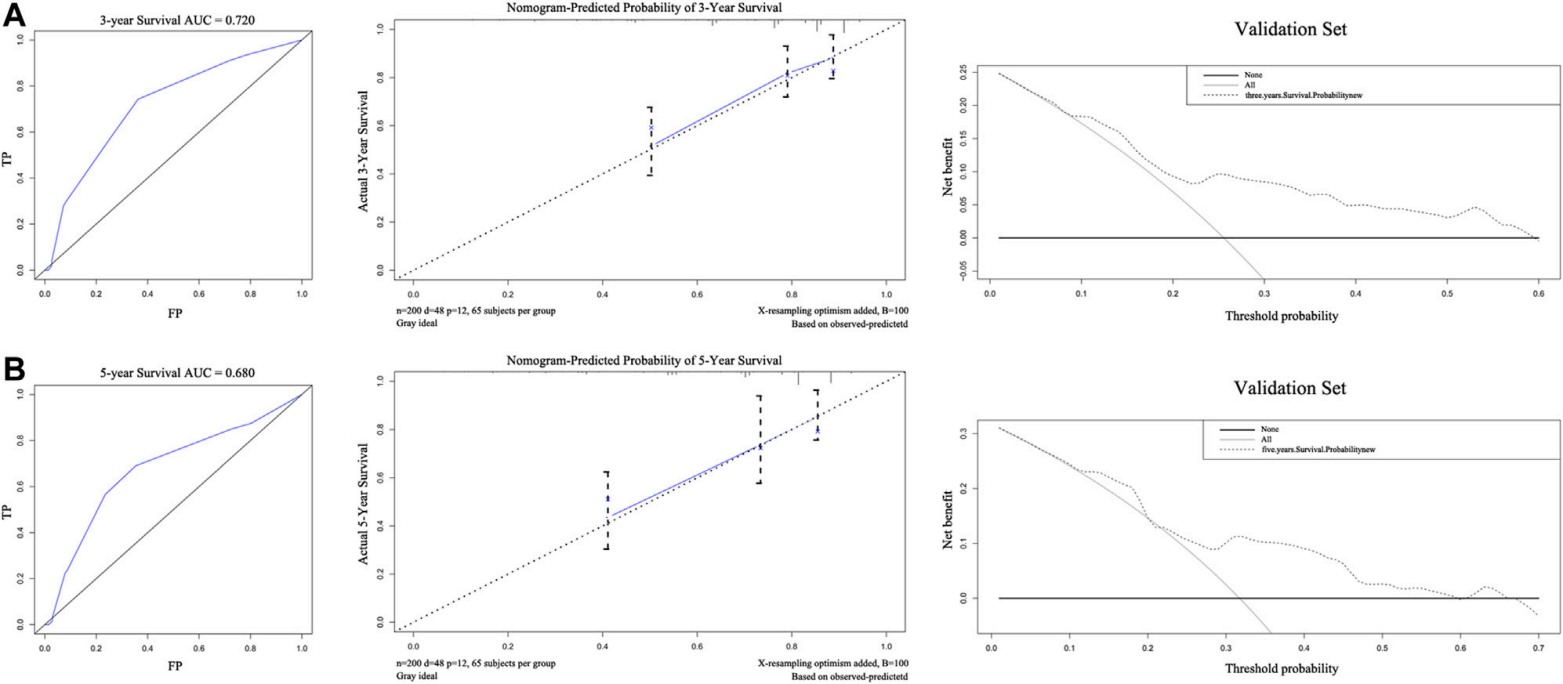
The areas under the receiver operating curves, Calibration plots, and decision curve analysis of OS associated nomograms in validation set. The validation set of 3-years **(A)** and 5-years **(B)**, respectively. OS, overall survival.

The external validation of the nomogram is illustrated in Figures 4A,B. The nomogram was used to assess each patient in the validation cohort. The C-index of the nomogram was 0.735. The ROC curves of 3-years and 5-years survival were 0.720 and 0.680, respectively. The calibration curves for 3-years and 5-years survival probabilities demonstrated good consistency between predicted results and actual observations. The DCA of 3-years and 5-years survival, within the threshold probability range of approximately 0.1–0.6 and 0.1–0.7, showed that the clinical utility of the nomogram is better than all-treat or none-treat scheme.

### Functional Enrichment Analyses of High- and Low-Risk Groups via GSEA

**FIGURE 5:**
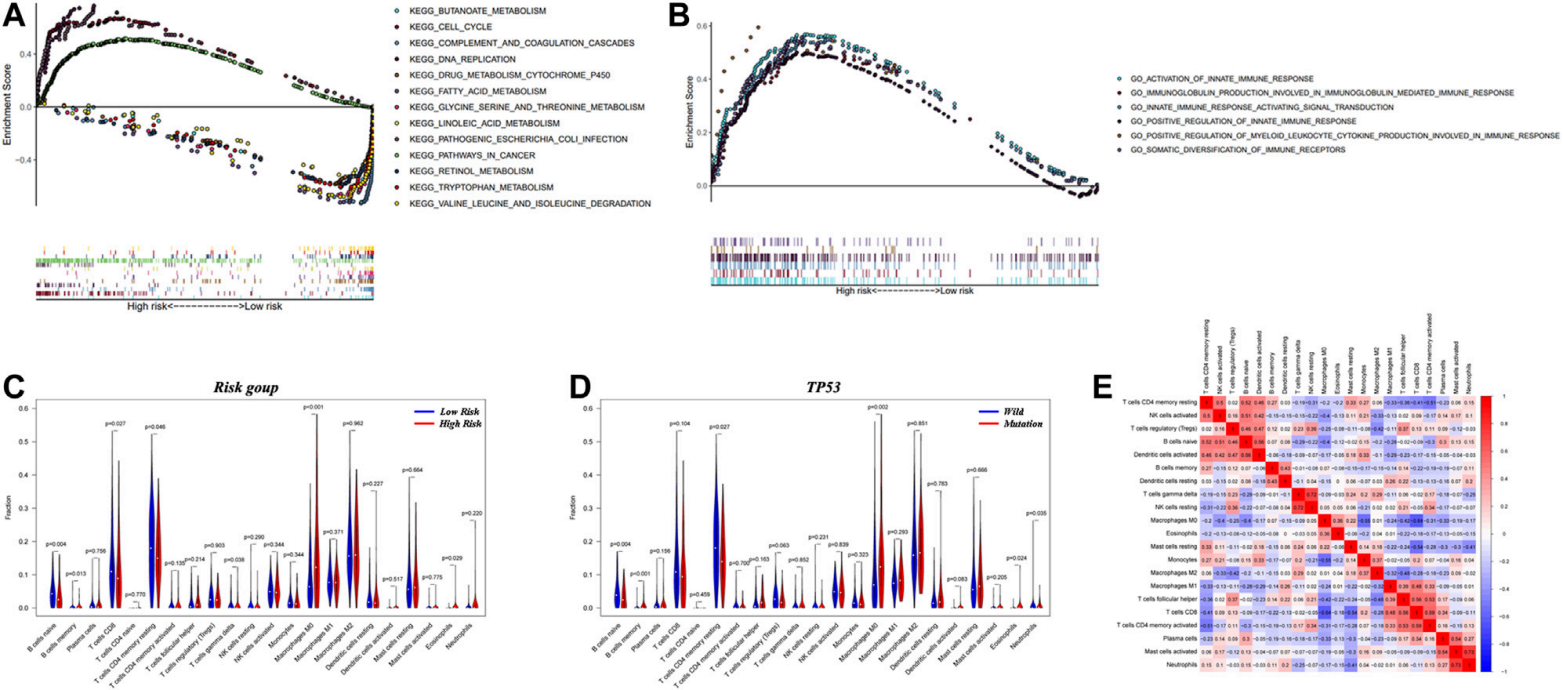
The results of gene set enrichment analysis based on Kyoto Encyclopedia of Genes and Genomes (KEGG) and Gene Ontology (GO) databases and the assessment of infiltrated immune cells. **(A)** Four significantly enriched KEGG pathways in high-risk group. Nine significantly enriched KEGG pathways in low-risk group. **(B)** Six significantly enriched GO pathways in high-risk group. **(C)** Relative proportion of immune cell infiltration in high- and low-risk patients. **(D)** Relative proportion of immune cell infiltration in TP53 mutation and normal status patients. **(E)** Correlation matrix of all 21 immune cell proportions. The red color represents positive correlation and the blue color represents negative correlations.

HCC patients from the TCGA cohort with complete immune therapy data (*n* = 219) were included for further study. Based on the nomogram-generated risk score, the cohort was separated into 88 high-risk subjects and 131 low-risk subjects. KEGG enrichment analysis showed that genes that characterized the high-risk group were mainly associated with responses to infection with pathogenic *Escherichia coli*, the cell cycle, DNA replication and cancer-related pathways. Genes characterizing the low-risk group, on the other hand, were found to be closely related to complement and coagulation cascades; metabolism of retinol, butanoate and fatty acids, including linoleic acid; metabolism of several amino acids, including tryptophan, glycine, and leucine; and drug metabolism through the cytochrome P450 pathway (Figure 5A).

GO enrichment analysis demonstrated that differentially expressed genes of the high-risk group were connected with activation of the innate immune response, production of immunoglobulins involved in immunoglobulin-mediated immune responses, signal transduction cascades activated during the innate immune response, positive regulation of the innate immune response, positive regulation of myeloid leukocyte cytokine production that facilitates immune responses and somatic diversification of immune receptors (Figure 5B), which suggested that the strong immune-related process might occur in tumor microenvironment of high-risk group patients.

### Connections Between Immune Cell Infiltration and the Nomogram

The close correlation between the high-risk group and immune-related biological pathways suggested potential biological mechanisms leading to poor outcomes in HCC. The distribution of the abundances of a variety of immune cells between high- and low-risk groups are displayed in Figure 5C. The high-risk group had a higher proportion of M0 macrophages and eosinophils. In contrast, the low-risk group had higher populations of naïve B cells, CD8^+^ T cells and resting CD4^+^ memory T cells. The relationships between these different immune cells and specific gene mutations is shown in Figure 5D, Supplementary Figure S1. As shown, mutations in TP53 were associated with a higher proportion of memory B cells, M0 macrophages and eosinophils. On the contrary, a normal TP53 status was associated with a higher population of naïve B cells and resting CD4^+^ memory T cells. The most obvious results in a heatmap of correlations (Figure 5E) were negative correlations between M0 macrophages and CD8^+^ T cells (correlation index = −0.64) and positive correlations between resting natural killer cells and gamma delta T cells (correlation index = 0.72).

### Prediction of Response to ICIs Treatment Based on TIDE and IPS

**FIGURE 6:**
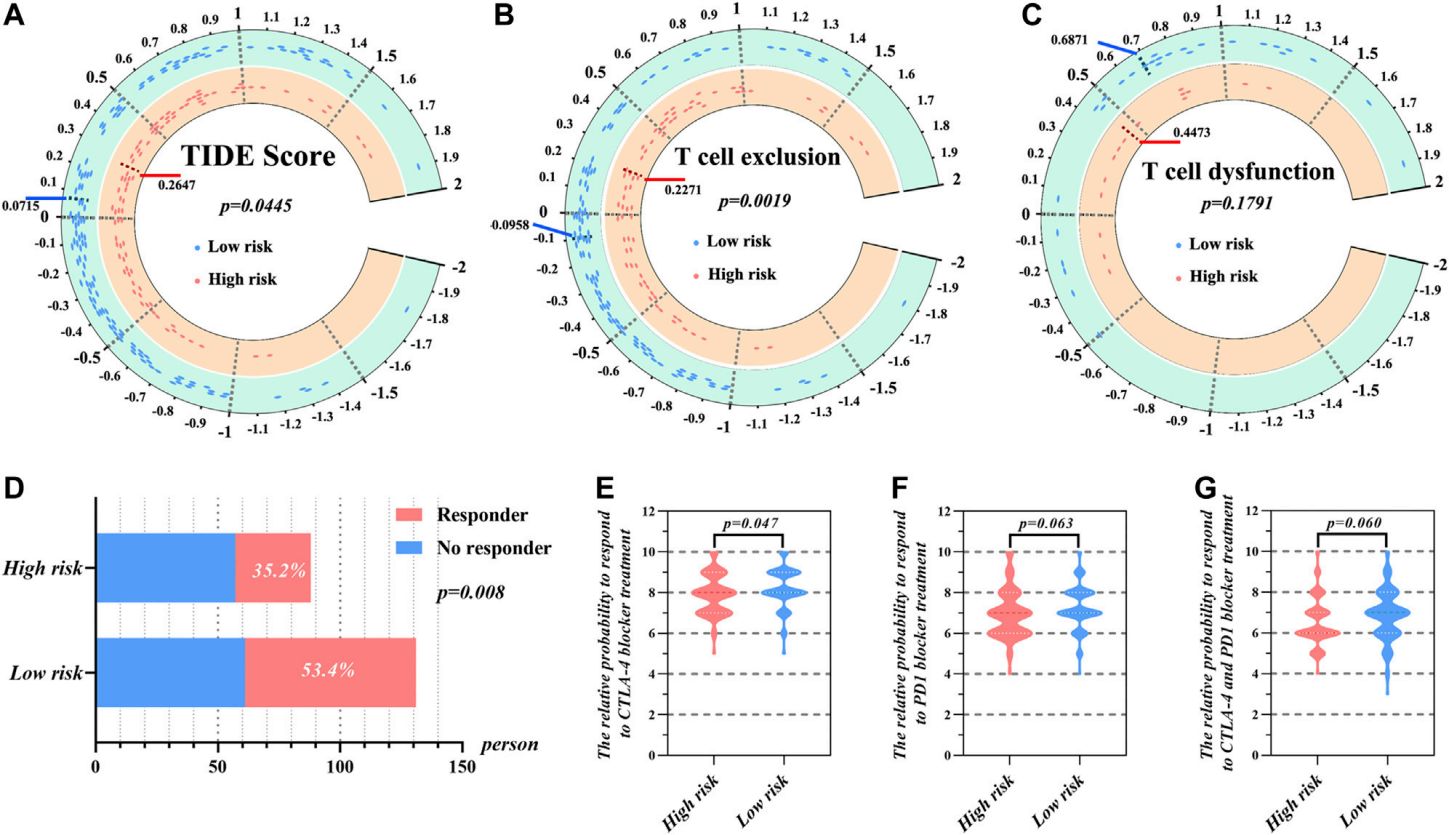
The prediction of response to immunotherapy of HCC patients in high- and low-risk group according to risk score. **(A)** The distribution of TIDE score, **(B)** T cell exclusion score, and **(C)** T cell dysfunction score in high- and low-risk groups. **(D)** The comparison of beneficiaries from immunotherapy between the high- and low-risk group. The relative probabilities of respond to CTLA-4 blocker treatment **(E)**, PD-1 blocker treatment **(F)** and the combination of CTLA-4 and PD-1 blocker treatment **(G)** in the low- and high-risk group.

As shown in Figure 6A, TIDE scores were significantly different between the high- and low-risk groups (*p* = 0.0445). The low-risk group had lower TIDE, which suggests that these patients would be more responsive to immune therapy. Furthermore, the high-risk group was characterized by a significantly higher T cell exclusion score than low-risk group (Figure 6B). However, there was no significant difference between high- and low-risk groups with regard to the T cell dysfunction score (Figure 6C). Further results showed that 53.4% patients from the low-risk group responded to immune therapy (Figure 6D), whereas only 35.2% patients from the high-risk group responded to immune therapy. This difference was statistically significant (*p* < 0.05).

We additionally utilized IPS to uncover the specific immune therapies that would have differential applicability to the high- and low-risk groups. The results demonstrated that responses to treatment with CTLA-4 blockers were significantly different between the high- and low-risk groups (Figure 6E). Here, the median IPS of the high-risk group was 7.920, while that of the low-risk group was 8.198. On the other hand, there was no significant difference between the high- and low-risk group when it came to PD-1 blocker treatment or treatment combining PD-1 and CTLA-4 blockers (Figures 6F,G).

## Discussion

The connections of mutations to cancer development and progression have led to advances in treatment, as various targeted therapies have focused on specific gene mutations (Maemondo et al., 2010; Zhao et al., 2017; Christensen et al., 2020). However, few studies have taken advantage of these connections in a predictive way by aiming to develop nomograms based on specific genes to guide the prognosis of HCC (Hsu et al., 2016; Wang et al., 2020). Our development of a strongly correlative predictive tool, then, fills an important gap in management of a common and deadly cancer. Moreover, given that the prognosis of HCC patients is affected by socioeconomic conditions and lifestyle, it is important to note that we have pioneered the inclusion of nationality as a prognostic factor into the model (Makarova-Rusher et al., 2016; Siegel et al., 2019). Our purposeful study of five different cohorts from four different countries strengthens clinical applications of our model by avoiding a narrow focus only on biological factors.

Considering the status of the majority of HCC cases, we paid specific attention to the population without distant or lymphatic metastasis. After a series of steps, we developed a model that used T stage, age, country and the mutation status of four specific genes (TP53, MACF1, EYS and DOCK2) as independent variables in the nomogram.

Given that so many variables, we used PCA to reduce the dimensionality of nomogram and achieved the visualization of samples distribution. Obviously, patients in the high- and low-risk groups are easy to distinguish in Figure 2C. SVM’s excellent discrimination between normal tissues and tumor tissues proved that these genes were of great significance for the occurrence and development of liver cancer. Mutations in both tumor protein 53 (TP53) and dedicator of cytokinesis 2 (DOCK2) were found to correlate with high risk and thus were considered to be antioncogenes. TP53 variants were universal and detected in 79.54% of Iranian lung cancer cases (Fathi et al., 2018). If the function of the TP53 gene product is lost due to mutation, the cell may lose regulation of growth, apoptosis and DNA repair (Hollstein et al., 1991; Blandino and Di Agostino, 2018). Accordingly, it has been reported that mutations in TP53 correlate with a worse prognosis after resection of colorectal liver metastases (Chun et al., 2019; Kawaguchi et al., 2019; Berg et al., 2020). Mutations in TP53 further influenced our prognostic model for HCC through alteration of immune-related genes. TP53 mutations have previously been shown to induce such alteration of immune-related genes (Long et al., 2019). Moreover, TP53 was involved in gene mutation classifier constructed by Luo et al. to guide the ICIs treatment for bladder cancer patients from Memorial Sloan Kettering Cancer Center, TCGA and other cohorts (Pan et al., 2021). Here, in the evaluation of immune cell infiltration, the fraction of M0 macrophages in the infiltrate were significantly higher in the TP53 mutation group, which suggests that mutations might induce the absence of CD8^+^ T cell and activated natural killer cells (Figures 5D,E). DOCK2, which belongs to the dedicator of cytokinesis protein family, plays an important role in migration, activation and proliferation of lymphocytes (Nishikimi et al., 2013; Jing et al., 2019). Patients with DOCK2 mutations have been shown to be more susceptible to immunodeficiency diseases (Dobbs et al., 2015). On the other hand, DOCK2 acted as an important participant in 4-gene signature for hypermutated colorectal cancer to identify suitable patients for immunotherapy (Ge et al., 2019).

Products of other mutated genes have intriguing but perhaps less direct connections to HCC. Mutations in another gene in our model, eyes shut homolog (EYS), have mainly been connected to ophthalmologic diseases, such as retinitis pigmentosa (Abd El-Aziz et al., 2008; Messchaert et al., 2018). The EYS protein is critical for protecting the stability of the ciliary axoneme in both rods and cones (Alfano et al., 2016). At present, the potential mechanisms linking EYS to oncogenic progression is still under development. Microtubule-actin crosslinking factor 1 (MACF1), is known to play an important role in regulating cytoskeleton dynamics, cell migration, growth and differentiation, and its abnormal expression has been closely connected to schizophrenia, Parkinson’s disease, cancer and osteoporosis (Hu et al., 2017). When HepG2 cells were transfected with the gene coding for hepatitis B protein X, the levels of the MACF1 protein varied, suggesting that MACF1 might play an important role in occurrence of liver cancer (Feng et al., 2010). Meanwhile, MACF1 mutation was used to distinguish three different immunotypes of muscle-invasive bladder cancer which were associated with benefit from ICIs (Chen et al., 2021). Importantly, our nomogram analysis combined critical genetic components with clinical and socioeconomic factors. It is clear that cancer occurrence and progression are impacted by more than just molecular biological influences, and predictive models should take into account these other factors. Here, it was not surprising to see that higher T stage and age correlated with worse prognoses. We were interested to see that country of residence was a specific and powerful risk factor. Country of residence likely represents a specific risk factor because it is related to lifestyle and socioeconomic conditions of patients.

The quality assessment and validation tests showed that the gene mutation-associated nomogram possesses excellent accuracy and therefore extensive clinical applications. For both the training and validation sets, the C-index of the nomogram was more than 0.70, which indicates that it has a high discrimination ability. The calibration curves for the 3-years and 5-years survival probabilities displayed good fitness between the predicted and actual observations. In addition, DCA indicated that the clinical utility of the nomogram significantly exceeded an all-treat or none-treat scheme. Hence, it is reasonable to predict the prognosis of HCC patients at early stage via the nomogram.

Because of limitations of the ICGC database, further functional analyses, such as GSEA, immune cell infiltration and prediction of response to ICIs treatments, were based on data from the TCGA database. The patients in the TCGA cohort were divided into high- and low-risk groups according to the nomogram risk score. GSEA analyses suggested that nomogram-based grouping resulted in accurate discrimination. Predicted KEGG pathways that have beneficial functions were enriched in the low-risk group. These pathways include those involved in metabolism of fatty acids, tryptophan, and retinol. Compared with high-risk group, the results indicated that tumor cells in low-risk population might possess higher differentiated grade so that they were qualified to perform normal physiological functions. On the other hand, the pathways enriched in high-risk group tended to involve the cell cycle and DNA replication. These cells, then, might be more prone to escape cell cycle checkpoints and to develop mutations that would enhance aberrant proliferation or other functions supporting malignant progression. Moreover, as there were numerous immune-related pathways in both high- and low-risk groups, we believe that the nomogram and the underlying findings may be applicable to the fine-tuning of our understanding of tumor immunity.

The results of infiltrated immune cell calculations indicated that there were more M0 macrophage cells recruited into the tumor microenvironment of the high-risk group and that this increase was accompanied by a significant decrease of CD8^+^ T cells. CD8^+^ T cells are the ultimate executors of the immune system in the destruction of tumor cells, via interaction with the T cell receptor (van der Leun et al., 2020). Accordingly, an absence of CD8^+^ T cells is an omen that the tumor immune microenvironment has deteriorated (Cheng et al., 2014). As associated immune surveillance weakens, a carcinoma is more apt to travel to other parts of the body in a phenomenon known as immune escape (Wu et al., 2021). Given the correlation of risk-score with CD8^+^ T cell status and the importance of these cells in immune escape, we employed a TIDE algorithm further focused on CD8^+^ T cell status. Where the degree of infiltration of CD8^+^ T cells was low, the score of T cell exclusion was noted (Figure 6B). The results of this analysis indicated that the immune microenvironment of high-risk patients was not conducive for treatment with ICIs, as these patients did not receive benefits of these inhibitors. Correspondingly, the analyses that compared IPS suggested that patients in the low-risk group were more likely to benefit from CTLA-4 blocker treatment. Thus, the results of TIDE and IPS analyses both indicate that the nomogram is applicable to guiding targeted immunotherapies.

While we have developed a powerful nomogram, there are some limitations that must be acknowledged. First, the study examined a modest number of patients; though it should be noted that the polycentricity of the sources provides a significant benefit. Second, we acknowledge the potential for selection bias, in that only patients with complete biological and clinical data were included. Finally, and most importantly, the potential prognostic factors available in public databases are finite. Further analysis with more complete data sets may enhance the predictive power of this tool.

## Conclusion

In this study, we combined multiple cohorts to established a nomogram based on gene mutations and clinical parameters. The clinical function of this nomogram involves more than just prognosis; the analysis extends to guidance of immunotherapy.

## Data Availability

The datasets presented in this study can be found in online repositories. The names of the repository/repositories and accession number(s) can be found in the article/Supplementary Material.
