# Supplementary material for: Development and Validation of a Gene Mutation-Associated Nomogram for Hepatocellular Carcinoma Patients From Four Countries
Source: Front Genet. 2021 Sep 21;12:714639. doi: 10.3389/fgene.2021.714639 (PMC8490742; doi:10.3389/fgene.2021.714639)
Supplement: Supplementary file 1 [file Table1.docx]

| Supplementary Table 1 Univariate cox analysis of 116 mutant genes. | | | | |
| --- | --- | --- | --- | --- |
| Mutation | HR | HR.95L | HR.95H | pvalue |
| TP53 | 1.497041184 | 1.021852879 | 2.19320447 | 0.03836665 |
| TTN | 0.896317735 | 0.58878949 | 1.36446981 | 0.609679927 |
| MUC16 | 1.155611828 | 0.709067687 | 1.88337266 | 0.561669439 |
| IGFN1 | 0.942166141 | 0.232761445 | 3.81367729 | 0.933445324 |
| AHNAK2 | 1.150751566 | 0.562067099 | 2.35599837 | 0.700922668 |
| MUC17 | 1.451599359 | 0.592211893 | 3.55808575 | 0.415252306 |
| OBSCN | 1.029249227 | 0.553726283 | 1.91313651 | 0.92737361 |
| TCHH | 0.944313824 | 0.300087365 | 2.9715633 | 0.921964293 |
| FLG | 0.625511527 | 0.255530216 | 1.5311875 | 0.304322409 |
| FLG2 | 1.736275689 | 0.426862779 | 7.06234747 | 0.440852611 |
| KMT2C | 0.433980388 | 0.107015101 | 1.75992898 | 0.24255987 |
| PCLO | 1.472963843 | 0.892329856 | 2.43141308 | 0.129905744 |
| ZAN | 1.380795517 | 0.438792788 | 4.3450948 | 0.581190111 |
| AHNAK | 0.338984332 | 0.047362105 | 2.42620927 | 0.281340875 |
| FAM186A | 0.674356414 | 0.094119554 | 4.83169068 | 0.694947407 |
| LRP1B | 0.763621503 | 0.372583587 | 1.56506572 | 0.46138524 |
| APOB | 0.982738322 | 0.55328015 | 1.74554357 | 0.952628446 |
| HMCN1 | 0.912308864 | 0.425388112 | 1.95658374 | 0.813619301 |
| TRIOBP | 1.692835186 | 0.537635672 | 5.33017267 | 0.368373937 |
| DNAH5 | 1.606035367 | 0.814160159 | 3.16811081 | 0.171682971 |
| MUC2 | 1.444969986 | 0.532940928 | 3.91776677 | 0.469497539 |
| ADGRV1 | 0.993840933 | 0.50421099 | 1.95894143 | 0.985763004 |
| SYNE1 | 0.581753717 | 0.214731611 | 1.57609486 | 0.286745508 |
| NEB | 0.533515345 | 0.169578456 | 1.67850698 | 0.282669156 |
| USH2A | 0.996213591 | 0.485915042 | 2.04241778 | 0.991736757 |
| XIRP2 | 1.02528457 | 0.5006556 | 2.09966382 | 0.945565949 |
| CSMD3 | 0.620240805 | 0.272965587 | 1.40933024 | 0.254031015 |
| RYR3 | 1.324590321 | 0.58300753 | 3.00946288 | 0.501995127 |
| HRNR | 1.371627017 | 0.191458907 | 9.82644632 | 0.75311493 |
| MACF1 | 2.03830339 | 1.032769248 | 4.02285478 | 0.040080583 |
| RYR2 | 1.52186023 | 0.872426538 | 2.6547319 | 0.139080942 |
| DNAH8 | 1.570837917 | 0.73157967 | 3.37288181 | 0.24673462 |
| DST | 1.298919476 | 0.632951405 | 2.66559453 | 0.475825896 |
| MDN1 | 1.475650578 | 0.602439112 | 3.61454724 | 0.394622667 |
| FSIP2 | 1.003367569 | 0.408454544 | 2.46476993 | 0.99415024 |
| CSMD1 | 1.547037218 | 0.833185264 | 2.87249938 | 0.166983721 |
| LRP2 | 1.092211919 | 0.4030086 | 2.96005315 | 0.862338386 |
| FAT3 | 1.279762455 | 0.670111241 | 2.44405979 | 0.454900741 |
| FRAS1 | 0.745650385 | 0.236741723 | 2.34852771 | 0.6160917 |
| DNAH14 | 1.466945318 | 0.466249879 | 4.61539758 | 0.512326205 |
| RYR1 | 0.922749978 | 0.376845522 | 2.25946037 | 0.86032631 |
| DNAH9 | 1.457723847 | 0.710934181 | 2.98896701 | 0.303617999 |
| LAMA1 | 0.770722345 | 0.245027387 | 2.42427159 | 0.656019399 |
| SMG1 | 1.09E-07 | 0 | Inf | 0.993949659 |
| RNF213 | 0.675274443 | 0.166801427 | 2.73376303 | 0.582084313 |
| PKHD1L1 | 0.519675729 | 0.165276952 | 1.63400196 | 0.262772322 |
| ANKRD36C | 1.10E-07 | 0 | Inf | 0.994994524 |
| EYS | 0.21189442 | 0.052377422 | 0.85722518 | 0.029554891 |
| CTNNB1 | 1.177069519 | 0.806700126 | 1.71748164 | 0.397724582 |
| ALB | 0.773052761 | 0.415951431 | 1.43673162 | 0.415635318 |
| ARID1A | 1.409400281 | 0.821526924 | 2.41794772 | 0.212726927 |
| AXIN1 | 1.195307589 | 0.626105568 | 2.28197976 | 0.588686263 |
| ARID2 | 1.073994807 | 0.562612356 | 2.05019466 | 0.828677153 |
| NFE2L2 | 1.756198384 | 0.891075883 | 3.46124592 | 0.103776465 |
| DNAH7 | 1.845895334 | 0.965117338 | 3.5304822 | 0.063932492 |
| PCDH15 | 1.36054809 | 0.71290533 | 2.59654547 | 0.350455713 |
| COL11A1 | 1.00208707 | 0.467353335 | 2.14864947 | 0.995725499 |
| FBN2 | 1.056744174 | 0.515738379 | 2.1652611 | 0.880134061 |
| MAP1B | 2.096174398 | 0.975924521 | 4.50234318 | 0.057763991 |
| ASPM | 0.897133592 | 0.125132777 | 6.43195732 | 0.91399026 |
| KMT2D | 1.537360421 | 0.676129416 | 3.49559864 | 0.304822381 |
| PREX2 | 1.399333194 | 0.516043884 | 3.79450944 | 0.509156959 |
| ATM | 0.959599996 | 0.353406341 | 2.60559035 | 0.935509045 |
| PRUNE2 | 0.923785989 | 0.341016634 | 2.5024602 | 0.876100906 |
| SPTA1 | 1.033704619 | 0.481644449 | 2.21853536 | 0.932202435 |
| SYNE2 | 0.586838109 | 0.186488314 | 1.84665172 | 0.362148048 |
| TNXB | 1.356737713 | 0.430076207 | 4.28002571 | 0.602738225 |
| RPS6KA3 | 1.105112672 | 0.451163282 | 2.70694462 | 0.826913647 |
| FREM1 | 1.351245149 | 0.498010072 | 3.66631833 | 0.554461576 |
| HYDIN | 1.396092775 | 0.5148579 | 3.78565627 | 0.51207675 |
| VPS13B | 0.999391457 | 0.368759149 | 2.70849764 | 0.999045194 |
| DYNC1H1 | 1.725000323 | 0.636315803 | 4.6763354 | 0.283930179 |
| MYCBP2 | 1.915929025 | 0.781601835 | 4.6964885 | 0.155223584 |
| COL22A1 | 0.529844319 | 0.130955408 | 2.14374501 | 0.37310569 |
| DCHS2 | 1.212477818 | 0.495347838 | 2.96781846 | 0.673138745 |
| FAT4 | 1.389060174 | 0.646545365 | 2.98430438 | 0.399650838 |
| ABCA13 | 1.575182387 | 0.84721767 | 2.92864472 | 0.151007535 |
| NBEA | 1.030772603 | 0.480727067 | 2.21017752 | 0.937923792 |
| ZFPM2 | 0.822806837 | 0.303465143 | 2.23093528 | 0.701545761 |
| CSMD2 | 1.220951654 | 0.498771498 | 2.98878935 | 0.662070956 |
| PDE4DIP | 0.969696864 | 0.396308577 | 2.3726764 | 0.94626088 |
| DNAH17 | 1.188148923 | 0.553747059 | 2.54935505 | 0.65806378 |
| TRRAP | 0.943772933 | 0.348419613 | 2.55642138 | 0.909377816 |
| DCC | 1.183012566 | 0.520282308 | 2.68992182 | 0.688420862 |
| COL6A3 | 0.440682113 | 0.108921155 | 1.78294772 | 0.250526634 |
| SETDB1 | 1.655444187 | 0.609691394 | 4.49488952 | 0.322627062 |
| HUWE1 | 1.190872225 | 0.439187644 | 3.22909052 | 0.731424412 |
| COL6A5 | 1.99380561 | 0.972233379 | 4.08879277 | 0.05968443 |
| VCAN | 1.404262195 | 0.518185677 | 3.80549367 | 0.504466819 |
| CTNNA2 | 0.459271777 | 0.113548676 | 1.85762242 | 0.27511584 |
| TSC2 | 1.659408688 | 0.770391636 | 3.57433423 | 0.195783846 |
| LAMA2 | 0.667026578 | 0.246343053 | 1.80611733 | 0.425600513 |
| UBR4 | 0.748087109 | 0.104429181 | 5.35898415 | 0.77265606 |
| SACS | 0.485110368 | 0.119827085 | 1.96393052 | 0.310619186 |
| ACVR2A | 1.470263569 | 0.68537797 | 3.15398956 | 0.32226533 |
| NRXN1 | 0.680563957 | 0.216196391 | 2.14234519 | 0.510701547 |
| PCDH10 | 1.289322734 | 0.475630148 | 3.49505413 | 0.617467431 |
| SETD2 | 1.076350221 | 0.39533557 | 2.93049724 | 0.885519198 |
| MGAM | 0.906532332 | 0.223630516 | 3.67481542 | 0.890703833 |
| NPAP1 | 0.775538837 | 0.284704263 | 2.11257984 | 0.61906931 |
| FASN | 1.037425041 | 0.423273399 | 2.54268451 | 0.935976293 |
| HERC2 | 1.314350927 | 0.61277221 | 2.81918522 | 0.482644302 |
| MUC4 | 1.422112921 | 0.720838068 | 2.80563035 | 0.309746584 |
| CACNA1E | 1.220586394 | 0.450112229 | 3.30991039 | 0.695334366 |
| DOCK2 | 3.312463684 | 1.861076616 | 5.8957356 | 4.67E-05 |
| CCDC168 | 1.30813889 | 0.534326902 | 3.20258507 | 0.556540181 |
| UNC80 | 1.4782657 | 0.688633016 | 3.17334404 | 0.31593528 |
| CUBN | 1.087743637 | 0.507154674 | 2.33298889 | 0.828961112 |
| MUC5B | 1.912318978 | 0.891448602 | 4.10227114 | 0.095936526 |
| FREM2 | 2.21735077 | 1.124080794 | 4.37392442 | 0.021594946 |
| LRP1 | 0.75287514 | 0.27784219 | 2.04008245 | 0.576770968 |
| SDK1 | 1.739072219 | 0.810130452 | 3.73319158 | 0.155685885 |
| BAP1 | 0.285930551 | 0.039784997 | 2.0549525 | 0.213425482 |
| PTPRQ | 1.794486259 | 0.732783133 | 4.39445286 | 0.200690981 |
| DNAH6 | 0.525596916 | 0.12985423 | 2.12740176 | 0.367215234 |
| DMD | 1.368041564 | 0.504392602 | 3.71047814 | 0.538171648 |
