# Supplementary figures and images for: Development and Validation of a Gene Mutation-Associated Nomogram for Hepatocellular Carcinoma Patients From Four Countries
Source: Front Genet. 2021 Sep 21;12:714639. doi: 10.3389/fgene.2021.714639 (PMC8490742; doi:10.3389/fgene.2021.714639)

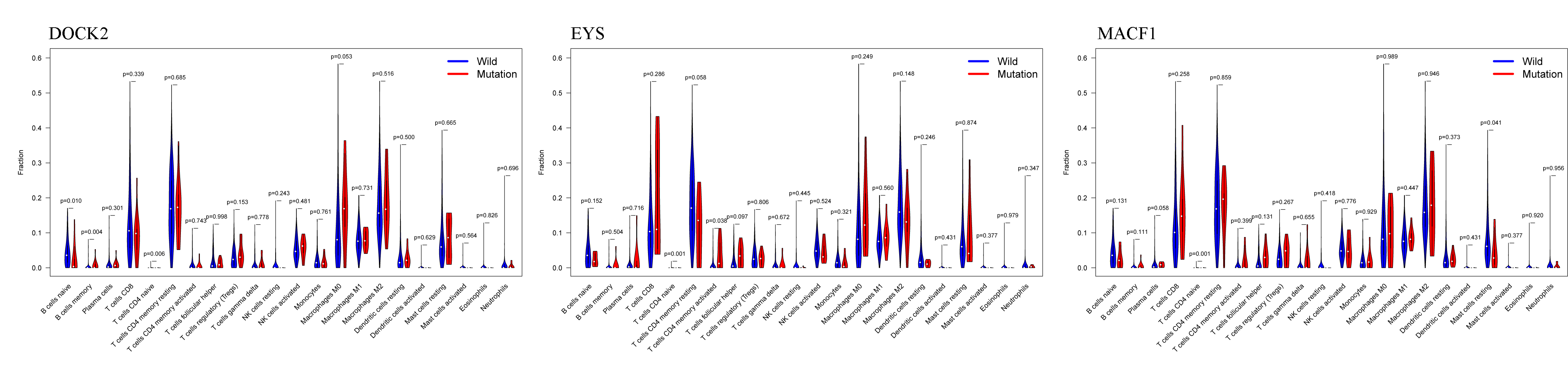

Supplement: Supplementary file 3 [file Image1.TIF]
