# Supplementary material for: Development and Validation of a Gene Mutation-Associated Nomogram for Hepatocellular Carcinoma Patients From Four Countries
Source: Front Genet. 2021 Sep 21;12:714639. doi: 10.3389/fgene.2021.714639 (PMC8490742; doi:10.3389/fgene.2021.714639)
Supplement: Supplementary file 4 [file Table2.docx]

| Supplementary Table 2 The progression of adjustment parameters for RBF nucleus. | | | |
| --- | --- | --- | --- |
| Ratio | Accuracy | Recall | AUC |
| 01:01.0 | 0.929688 | 0.99115 | 0.897935 |
| 01:01.1 | 0.929688 | 0.99115 | 0.888496 |
| 01:01.2 | 0.929688 | 0.99115 | 0.866667 |
| 01:01.3 | 0.929688 | 0.99115 | 0.840118 |
| 01:01.4 | 0.921875 | 0.99115 | 0.822419 |
| 01:01.5 | 0.921875 | 0.99115 | 0.816519 |
| 01:01.6 | 0.921875 | 0.99115 | 0.815929 |
| 01:01.7 | 0.921875 | 0.99115 | 0.813569 |
| 01:01.8 | 0.921875 | 0.99115 | 0.814159 |
| 01:01.9 | 0.914062 | 0.99115 | 0.813569 |
| 01:02.1 | 0.914062 | 0.99115 | 0.812979 |
| 01:02.2 | 0.914062 | 0.99115 | 0.812389 |
| 01:02.3 | 0.914062 | 0.99115 | 0.810619 |
| 01:02.4 | 0.914062 | 0.99115 | 0.80944 |
| 01:02.5 | 0.914062 | 0.99115 | 0.80885 |
| 01:02.6 | 0.914062 | 0.99115 | 0.80708 |
| 01:02.7 | 0.914062 | 0.99115 | 0.8059 |
