# Supplementary material for: Development and Validation of a Gene Mutation-Associated Nomogram for Hepatocellular Carcinoma Patients From Four Countries
Source: Front Genet. 2021 Sep 21;12:714639. doi: 10.3389/fgene.2021.714639 (PMC8490742; doi:10.3389/fgene.2021.714639)
Supplement: Supplementary file 5 [file DataSheet2.docx]

Supplementary files Code

1. Random Grouping

library(rms)

library(foreign)

library(survival)

setwd("D:/R work")

dev<-read.csv("dev.csv")

head(dev)

library(caret)

set.seed(131)

trianandvad<- createDataPartition(y=dev$id,p=0.70,list=FALSE)

train <- dev[trianandvad, ]

vadi<-dev[-trianandvad,]

write.csv(train, "train.csv")

write.csv(vadi, "vadi.csv")

2. Calcuating Risk Score

ibrary(survival)

setwd("C:\\Users\\4_ROC\\risk")

seer<-read.table("seer.txt",header=T,sep="\t",check.names=F,row.names=1)

cox_m <- coxph(Surv(survival_time, status) ~age + T + Country + TP53 + MACF1 + EYS + DOCK2, data = seer)

cox_m1<-step(cox_m,direction = "both")

risk_score<-predict(cox_m1,type="risk",newdata=seer)

risk_level<-as.vector(ifelse(risk_score>median(risk_score),"High","Low"))

write.table(cbind(id=rownames(cbind(seer[,1:2],risk_score,risk_level)),cbind(seer[,1:2],risk_score,risk_level)),"risk_score.txt",sep="\t",quote=F,row.names=F)

3.Python

import pandas as pd

import numpy as np

from sklearn.model_selection import train_test_split

from sklearn.svm import SVC

import matplotlib.pyplot as plt

liver = pd.read_csv(r"C:\Users\Desktop\vad\SVC.csv",index_col=0)

X = liver.iloc[:,:-1]

Y = liver.iloc[:,-1]

Xtrain, Xtest, Ytrain, Ytest = train_test_split(X,Y,test_size=0.3,random_state=420)

score = []

gamma_range = np.logspace(-10, 1, 50)

for i in gamma_range:

clf = SVC(kernel="rbf",gamma = i,cache_size=5000).fit(Xtrain,Ytrain)

score.append(clf.score(Xtest,Ytest))

print(max(score), gamma_range[score.index(max(score))])

plt.plot(gamma_range,score)

plt.show()

irange = np.linspace(0,2,20)

for i in irange:

clf = SVC(kernel = "rbf"

,gamma= 5.963623316594637

,cache_size = 5000

,class_weight = {1:1+i}

).fit(Xtrain, Ytrain)

result = clf.predict(Xtest)

score = clf.score(Xtest,Ytest)

recall = recall_score(Ytest, result)

auc = roc_auc_score(Ytest,clf.decision_function(Xtest))

print("under ratio 1:%f testing accuracy %f, recall is %f', auc is %f" %(1+i,score,recall,auc))
